# Telomere Shortening, Inflammatory Cytokines, and Anti-Cytomegalovirus Antibody Follow Distinct Age-Associated Trajectories in Humans

**DOI:** 10.3389/fimmu.2017.01027

**Published:** 2017-08-24

**Authors:** Ana Lustig, Hans B. Liu, E. Jeffrey Metter, Yang An, Melissa A. Swaby, Palchamy Elango, Luigi Ferrucci, Richard J. Hodes, Nan-ping Weng

**Affiliations:** ^1^Laboratory of Molecular Biology and Immunology, National Institute on Aging, National Institutes of Health, Baltimore, MD, United States; ^2^Department of Neurology, University of Tennessee Health Science Center, Memphis, TN, United States; ^3^Laboratory of Behavioral Neuroscience, National Institute on Aging, National Institutes of Health, Baltimore, MD, United States; ^4^Translational Gerontology Branch, National Institute on Aging, National Institutes of Health, Baltimore, MD, United States; ^5^Experimental Immunology Branch, National Cancer Institute, National Institutes of Health, Bethesda, MD, United States

**Keywords:** telomeres, IL-6, IL-10, interferon gamma, anti-CMV IgG, aging

## Abstract

A number of biological parameters have been cited as hallmarks of immune aging. However, it is not clear whether these multiple biological changes are the result of common underlying aging processes and follow correlated trajectories, or whether the patterns of change for multiple parameters vary across individuals and reflect heterogeneity in the aging process. Here, we have studied parameters of immune system aging through longitudinal analysis of telomere length, inflammatory cytokines, and antibody titer to cytomegalovirus (CMV) in 465 subjects ranging in age from 21 to 88 years at the first visit, with an average of 13 years (7–19 years) follow-up. We observed a highly variable rate of change in telomere length of PBMCs with a relatively slow average rate of telomere shortening (−16 bp/year). Similarly, there were significant increases with age *in vivo* in three inflammation-related cytokines (interferon gamma, IL-6, and IL-10) and in anti-CMV IgG titer, which varied widely across individuals as well. We further observed positive correlative changes among different inflammatory cytokines. However, we did not find significant correlations among the rate of changes in telomere length, inflammatory cytokines, and anti-CMV IgG titers. Our findings thus reveal that age-related trajectories of telomere attrition, elevated circulating inflammatory cytokines, and anti-CMV IgG are independent and that aging individuals do not show a uniform pattern of change in these variables. Immune aging processes are complex and vary across individuals, and the use of multiple biomarkers is essential to evaluation of biological aging of the immune system.

## Introduction

Decline in immune function with age is a major contributor to increased mortality and morbidity in older adults. The identification in the past decades of various age-associated changes within the immune system provides a foundation for understanding alteration of immune function in the elderly (1–4). The wide range of biomarkers that change with age include (1) telomere shortening (5–7), (2) increased circulating pro-inflammatory cytokines (8, 9), (3) alteration of cell subpopulation composition (reduced naive lymphocytes and increased viral specific effector cells) (10–12), and (4) reduced overall immune response to challenges such as vaccination (13, 14). It remains to be determined whether these age-associated changes occur *in vivo* in parallel or independently, or whether patterns of change are uniform or different across individuals. Addressing these questions is of importance in understanding how underlying mechanisms affect different aspects of human immune system aging.

Telomeres are the ends of linear chromosome and play an essential role in maintaining chromosome stability (15). Due to the inability of DNA polymerase to completely replicate chromosomal ends, loss of telomeres occurs after each cell division. Telomere length attrition with age in blood leukocytes and mononuclear cells (PBMCs) and in isolated T and B lymphocytes has been reported mainly from cross-sectional studies, with telomere attrition reported to be ~25 bp/year (16). A more limited number of studies with longitudinal follow-up of the same individuals have been reported (17, 18), and these studies show that the rate of telomere attrition with age is highly heterogeneous among individuals. The overall rates of telomere attrition from longitudinal studies appear higher than those reported in cross-sectional studies, ranging from −23 to −40 bp/year (16, 18). The presence of shorter telomeres in older populations is associated with reduced immune functions (19), but the factors contributing to telomere attrition with aging are not fully understood.

Increase of circulating pro-inflammatory cytokines in the old is a consistently reported age-associated change in the immune system (20, 21). Increase in IL-6, a multi-functional cytokine, is considered to be an important biomarker of aging and has been associated with the decline of immune function in older adults (22–25). Increase in interferon gamma (IFN-γ) is also reported from cross-sectional studies, but understanding of its trajectory is limited due to lack of longitudinal analysis. IL-10 is considered as an anti-inflammatory cytokine and is produced by both innate and lymphoid cells (26). In contrast to increases in IFN-γ and IL-6, it has been reported that serum IL-10 decreased in a 10-year follow-up longitudinal study (25). Although the levels of cytokine production are determined in part by genetic influences (27), the inter-relationship of age-related changes among different pro- and anti-inflammatory cytokines is less known.

Chronic infection with cytomegalovirus (CMV) has a profound impact on the immune system and is considered one of the causes of immunosenescence in the elderly (28). The serum titer of CMV-specific IgG has been widely used as an indicator of CMV infection status, but its significance in immunosenescence is less well defined (29). Age-associated increase in anti-CMV IgG has been reported from cross-sectional studies (29, 30), but its trajectory has not been analyzed in longitudinal studies. Although a recent study found no difference of telomere length in subjects between CMV seropositive and negative from a cross-sectional analysis (31), it is unknown whether the rates of changes of these age-associated biomarkers *in vivo* are correlated or independent.

In this study, we sought to measure the *in vivo* changes of telomere length, inflammation-related cytokine and anti-CMV antibody titer with age and to determine the trajectory of these age-associated immune changes and their inter-relationship using longitudinal analysis over an average of 13 years. Specifically, we assessed the individual longitudinal trajectories of PBMC telomere length, eight pro-inflammatory cytokines, and anti-CMV IgG titer in 456 subjects. Strikingly, aging-associated changes in these variables occur with a distinct trajectory in each individual. Thus, immune aging is a heterogeneous process across individuals, and an assessment of immunosenescence requires a combinatorial evaluation of multiple age-associated biomarkers.

## Materials and Methods

### Study Design and Participants

We performed a longitudinal study of 465 Baltimore Longitudinal Study on Aging (BLSA) participants at first visit and an average of 13 years follow-up (ranging from 7 to 18 years) under an IRB-approved protocol. Demographic characterization of these participants is summarized in Table S1 in Supplementary Material. At each visit, fasting blood was collected. Sera were isolated from blood stored in a −80°C freezer, and genomic DNA was isolated from PBMCs using the Qiagen kit (QIAamp DNA mini Kit) and stored at −80°C.

### Measurement of Telomere Length of PBMCs

The procedure for telomere length measurement by the terminal restriction fragment using Southern blot was previously described (32). DNA from Jurkat cells was loaded on every gel and used for normalization. The coefficient of variation of telomere length of Jurkat cells measured at different times was 10.6% (*n* = 146). The rates of telomere length change were calculated as (difference in telomere length between measurements) divided by (time between measurements) between samples.

### Measurement of Cytokines by MESO Kit

Serum was isolated from fasting blood that was collected at each visit and stored at −80°C prior to cytokine measurement. A total of 432 subjects, each with two visits separated by an average of 13 years, were tested for 10 human pro-inflammatory cytokines (IFN-γ, IL-1β, IL-2, IL-4, IL-6, IL-8, IL-10, IL-12p70, IL-13, and TNF-α) by Meso Scale Discovery assay (human pro-inflammatory panel 1 MSD Multi-spot assay system, Rockville, MD, www.mesoscale.com). Aliquots from the samples were added to plates pre-spotted with an array of antibodies to each of the 10 cytokines and allowed to bind at room temperature for 2 h. The plates were then washed, and a cocktail mix of the same antibodies linked to electrochemiluminescent tags was added and allowed to bind for 2 h. After washing the plates, a substrate buffer was added that allowed bound antibody to emit light when analyzed with an electric charge on the Meso Quickplex SQ120. The instrument measured and recorded the intensity of the emitted light from each spot in each well. Quantitative analysis was calculated against a standard curve for each cytokine using MSD discovery software provided by the company.

### Measurement of CMV IgG

Cytomegalovirus IgG was measured from sera of 352 subjects who had two visits separated by an average of 13 years, using the ELISA kit (Abcam, # ab108639) according to the manufacturer’s instructions. Sample values were normalized to the standard. For qualitative analysis, values below 1 were considered negative and above 1 considered as positive as determined by the standard.

### Statistical Analysis

Data presented in figures plotted the relationships between telomere length, cytokines, and CMV IgG by age. The relationship between the markers, age, and the longitudinal effect of time (rate) were tested using mixed effects linear regression on initial age and time (rate) between measurements with a random effect for subject to address the within-subject correlation with the repeated measurements (33). For models including the cytokines and CMV IgG, further adjustments were included for date. Interactions were examined between initial age and longitudinal time. For the regression models, all tests were performed with a *P* < 0.05 using lme4 (version 1.1-12) and lmerTest (2.0-33). Rates of change presented in Table 1 were evaluated for age group differences using ANOVA. Partial correlations were calculated between pairs of variables adjusted for sex and age with the cytokine and CMV data adjusted for date effects. *P* < 0.001 was considered significant at *P* < 0.05 based on multiple comparisons adjusted using false discovery rate using the tool FDRtool in R (34). All statistical analyses were done using R version 3.3.2 (http://www.r-project.org).

**Table 1:** Rate changes of telomere length, cytokines, and anti-CMV IgG in age groups.

Rate of change	Gender	Value	Age group						
			<40	40–49^a^	50–59	60–69	70–79	>80	*P*^b^
Telomere (bp/year)	Male	Mean	−39.9	−4.8	−5.6	−14.6	−20.9	−4.6	0.01
		SD	77.9	32.4	29.2	38.3	32.8	28.7	
	Female	Mean	−42.6	−13.8	−6.7	−20.6	−27.6	−29.6	0.003
		SD	65.0	34.5	34.1	30.9	39.6	35.4	
Interferon gamma (IFN-γ) (pg/mL/year)	Male	Mean		−0.17	0.12	0.12	−1.13	0.3	0.7
		SD		1.16	1.05	0.46	8.98	1.39	
	Female	Mean		0.13	0.07	0	−0.42	0	0.24
		SD		0.56	0.55	0.36	2.31	0.25	
IL-6 (pg/mL/year)	Male	Mean		0.07	0.52	0.14	0.08	0.25	0.67
		SD		0.16	2.69	0.27	0.27	0.29	
	Female	Mean		0.03	0.04	0.11	0.16	0.20	0.02
		SD		0.07	0.11	0.29	0.37	0.23	
IL-10 (pg/mL/year)	Male	Mean		0.004	0.012	0.024	0.016	0.059	0.61
		SD		0.024	0.068	0.087	0.034	0.150	
	Female	Mean		0.005	0.023	0.014	−0.024	0.014	0.73
		SD		0.025	0.142	0.051	0.224	0.044	
Anti-CMV IgG (U/mL/year)	Male	Mean		0.17	0.09	0.21	0.20	0.46	0.89
		SD		0.51	0.61	0.58	0.95	0.53	
	Female	Mean		0.20	0.24	0.20	0.38	0.31	0.94
		SD		0.60	0.74	0.64	0.75	0.59	

*^a^For rates of age group 40–49 years for IFN-γ, IL-6, IL-10, and anti-cytomegalovirus IgG included subjects under 40 years old*.

*^b^The P value is from an ANOVA model and addresses whether the age groups differ*.

## Results

### Change of Telomere Length in PBMCs with Age *In Vivo*

**Figure 1:**
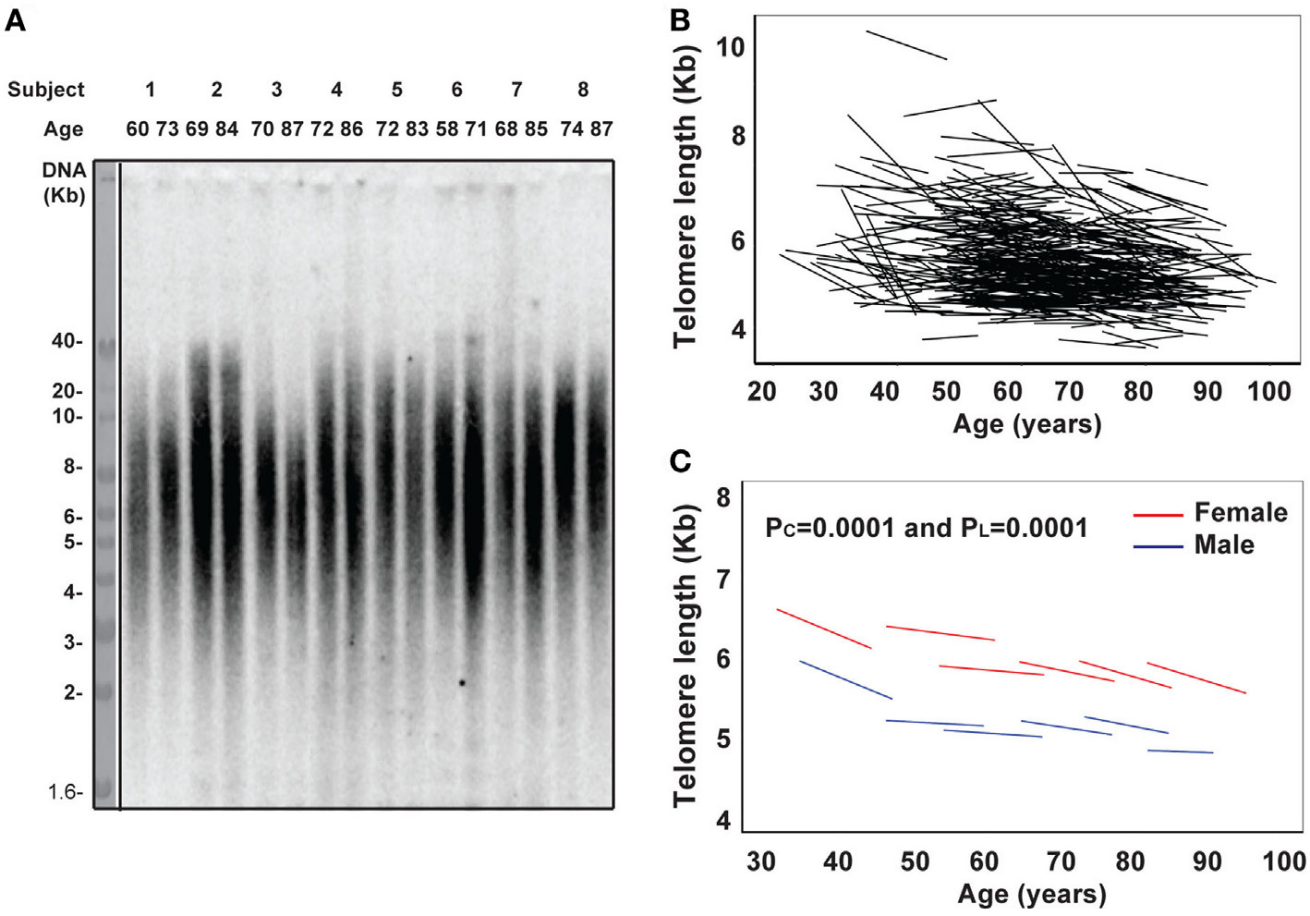
Reduction of telomere length in PBMCs with age *in vivo*. **(A)** Representative image of telomere measurement. DNA from PBMCs of eight subjects (age at each visit) was digested, separated on 0.6% agarose gel, and probed with ^32^P-TTAGGG_4_. The image was collected from a phosphorimager. **(B)** Graph of telomere length (kb) of PBMCs in each subject for the two visits connected by a line (*N* = 465). **(C)** Graph of telomere length (kb) of PBMC by six age groups (under 40, 40–49, 50–59, 60–69, 70–79, and 80 years and older) for male in blue and female in red. Two *P* values were presented: *P*_C_ refers to the initial evaluation and represents cross-sectional effect of age, and *P*_L_ refers to the longitudinal time change within subjects (for all figures). Models were calculated by linear mixed effect regression (33). *P* < 0.05 is considered as significant.

Here, we have analyzed 465 BLSA subjects aged 21–88 years at the first visit, with an average of 13 years (ranging from 7 to 18 years) follow-up (Table S1 in Supplementary Material). We measured telomere length of PBMCs by Southern blot and analyzed telomere length change individually as well as across six age categories: under 40 years (male = 19 and female = 23 subjects), 40–49 years (M = 32 and F = 58), 50–59 years (M = 65 and F = 66), 60–69 years (M = 64 and F = 43), 70–79 years (M = 47 and F = 25), and over 80 years (M = 13 and F = 11) at the first visit. Telomere length was highly variable among individuals within each age group, ranging from 3.5 to 11.3 kb (Figure 1A; Table S2 in Supplementary Material). The rate of telomere length change was calculated based on the difference between measurements over the time between visits and also exhibited a high degree of individual variation ranging from −304 to +58 bp/year (Figure 1A; Table 1).

Among the different age groups, the under 40 years age group had the highest mean rate of telomere attrition (−40 and −43 bp/year for men and women, respectively), whereas the middle age groups (40–69 years) had the overall lowest mean rates of telomere attrition (−9 and −13 bp/year for men and women, respectively). Interestingly, age group (70–79 years) had a higher rate of telomere attrition than the middle age groups but lower than the under 40 years age group (Figure 1B; Table 1). Overall, significant telomere attrition with age was observed from both cross-sectional analysis (*P*_C_ = 0.0001) and longitudinal analysis (*P*_L_ = 0.0001), and the average rate of telomere attrition in PBMCs with age was relatively slow (−16 bp/year) compared to the range of previous reports (16). In agreement with a previous report (35), we found that women had longer telomeres than men across all six age groups and the average difference was 0.6 kb. This gender difference was relatively stable (0.4–0.9 kb) throughout the adult ages.

### Increases in Circulating Inflammation-Related Cytokines (IFN-γ, IL-6, and IL-10) *In Vivo* with Age

**Figure 2:**
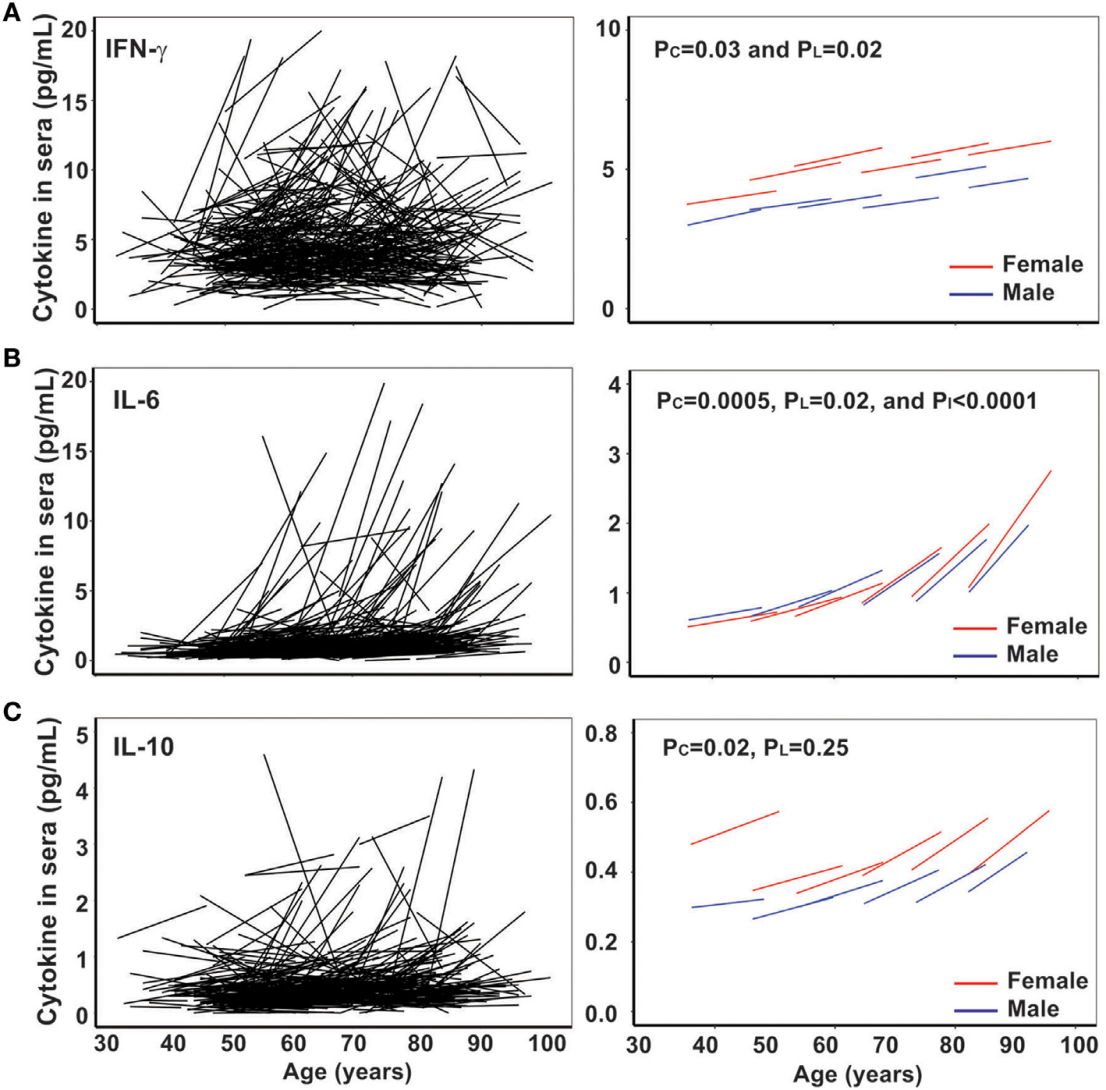
Increase of circulating interferon gamma (IFN-γ), IL-6, and IL-10 with age *in vivo*. **(A)** Line graphs of IFN-γ (pg/mL) by date adjusted raw data for the two visits of each subject (*N* = 432) (left) and by five age groups (under 50, 50–59, 60–69, 70–79, and 80 years and older) for male in blue and female in red (right). **(B)** Line graph of IL-6 (pg/mL) by date adjusted raw data for the two visits of each subject (*N* = 432) (left) and by five age groups (right). **(C)** Line graph of IL-10 (pg/mL) by date adjusted raw data for the two visits of each subject (*N* = 432) (left) and by five age groups (right). *P*_I_ refers to the age group difference using the mixed effects model.

Next, we analyzed another age-related parameter, circulating inflammatory cytokines (IL-1β, IL-2, IL-4, IL-6, IL-10, IL-12p70, and IFN-γ) in 432 BLSA subjects who had sera available from at least two visits. We found that levels of all inflammatory cytokines exhibited a wide range among the study subjects. We applied the mixed effects linear regression on initial age and time between measurements and found that three inflammatory cytokines (IFN-γ, IL-6, and IL-10) showed statistically significant age-related changes (Figure 2), while five inflammatory cytokines (IL-1β, IL-2, IL-4, and IL-12p70) did not (Figure S1 in Supplementary Material).

IFN-γ plays an essential role in immune response and inflammation. Increased circulating IFN-γ in the blood of older subjects compared to younger subjects has been previously reported (36), but the longitudinal trajectory with age has not been reported. In agreement with previous reports, we found the level of circulating IFN-γ increased with age from 7 and 5 pg/mL (male and female, respectively) in the under 50 years age group to 16 and 11 pg/mL (male and female, respectively) in the 70–79 years age group (Figure 2A left graph). The increase in circulating IFN-γ in blood with age was significant from both cross-sectional (*P*_C_ = 0.031) and longitudinal (*P*_L_ = 0.019) analyses (Figure 2A right graph). Interestingly, women appeared to have a higher average IFN-γ level than men.

IL-6 is a multi-functional cytokine, and its increase in circulation with age is considered as an important biomarker of aging, associated with predicted functional status (22–24). In agreement with previous findings, we found that the increase in IL-6 with age *in vivo* exhibited individual variation: from 0.07 and 0.03 pg/mL/year (male and female, respectively) in the under 50 years age group to 0.25 and 0.20 pg/mL/year (male and female, respectively) in the >80 years age group (Table 1). Significant increase in circulating IL-6 with age was demonstrated for both cross-sectional (age at first evaluation, *P*_C_ = 0.019) and longitudinal (change over time, *P*_L_ < 0.0001) coefficients using the linear mixed model (Figure 2B right graph). Strikingly, the rate of increase in circulating IL-6 displayed an increasing pace with advancing age (*P*_I_ < 0.0001) (Figure 2B right graph). Finally, the level and rate of change in IL-6 were comparable between men and women (Figure 2B), though age group differences were only significant for women (Table 1). In contrast to the reported reduction in IL-10 level with aging (25), we observed a significant increase in IL-10 with age of our study subjects from the cross-sectional analysis (*P*_C_ = 0.014) but not longitudinal analysis (*P*_L_ = 0.223) due partly to higher variability of the rates among the study subjects (Figure 2C; Table S2 in Supplementary Material). Together, these findings reveal that the rate of increase in IFN-γ and IL-10 did not change with age but that the rate of increase in IL-6 did increase with advancing age.

### Increase of Anti-CMV IgG Serum Titer with Age

**Figure 3:**
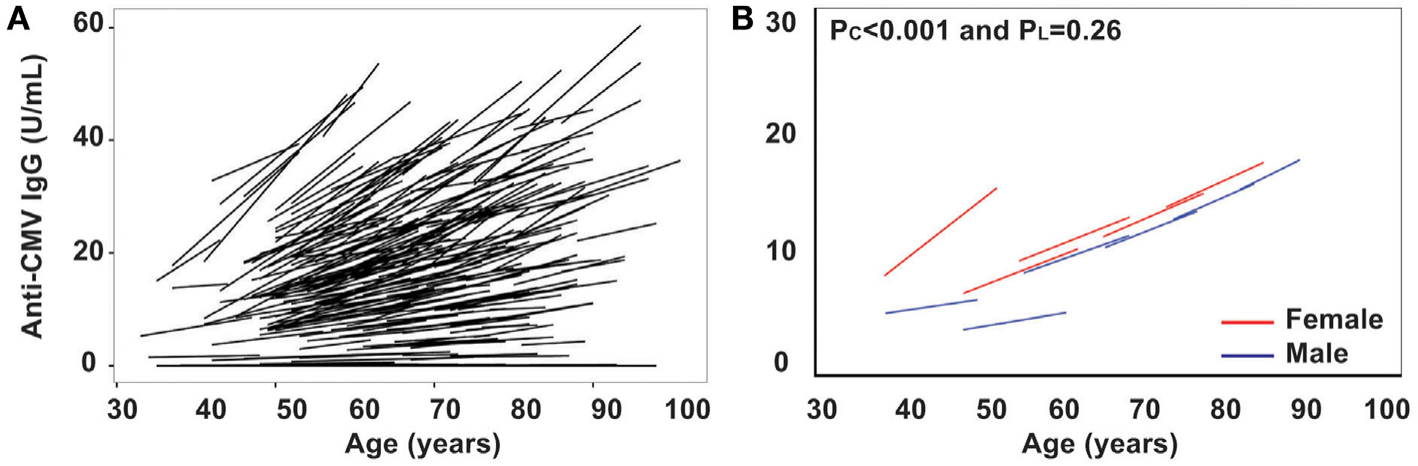
Increase in titers of anti-cytomegalovirus (CMV)-IgG in serum with age *in vivo*. **(A)** Line graph of anti-CMV-IgG for the two visits of each subject (*N* = 435). **(B)** Graph of anti-CMV-IgG by five age groups (under 50, 50–59, 60–69, 70–79, and 80 years and older) for male in blue and female in red.

Persistent CMV infection has a profound impact on the immune system, and the positivity of anti-CMV IgG in sera increases with age and is a marker of immune aging (28–30). However, the trajectory of the anti-CMV IgG titer with age has not been examined. Here, we measured anti-CMV IgG by ELISA in 417 subjects and found that there was a significant increase in anti-CMV IgG titer in serum with age: from 4.4 and 9.9 U/mL (male and female, respectively) in the under 50 years age group to 17 and 28 U/mL (male and female, respectively) in the over 80 years age group (Figure 3). In addition, the percentage of individuals positive for anti-CMV IgG increased with age: from 32 and 43% (male and female, respectively) of under 50 years age group to 70 and 82% (male and female, respectively) of over 80 years age group. Overall, significant increase in anti-CMV IgG titers was observed from the cross-sectional analysis (*P*_C_ < 0.001) but not from the longitudinal analysis (*P*_L_ = 0.26), potentially due at least in part to the shorter overall age range assessed in the longitudinal samples (Figure 3B). Nevertheless, the average rates derived from the cross-sectional and longitudinal analysis were comparable. The rates of anti-CMV IgG titer increase were relatively stable, with mild acceleration in the over 80 years age group when compared to the under 50 years age group (Figure 3B). Women exhibited an average higher anti-CMV IgG titer than men in all age groups (Table 1). These findings suggest that age-associated increase in serum anti-CMV IgG (both percentage of positivity and actual titers) exhibits a gender and age difference.

### Distinct and Independent Trajectories of Age-Associated Changes of Telomere Length, Inflammatory Cytokines, and Anti-CMV IgG Titer

**Figure 4:**
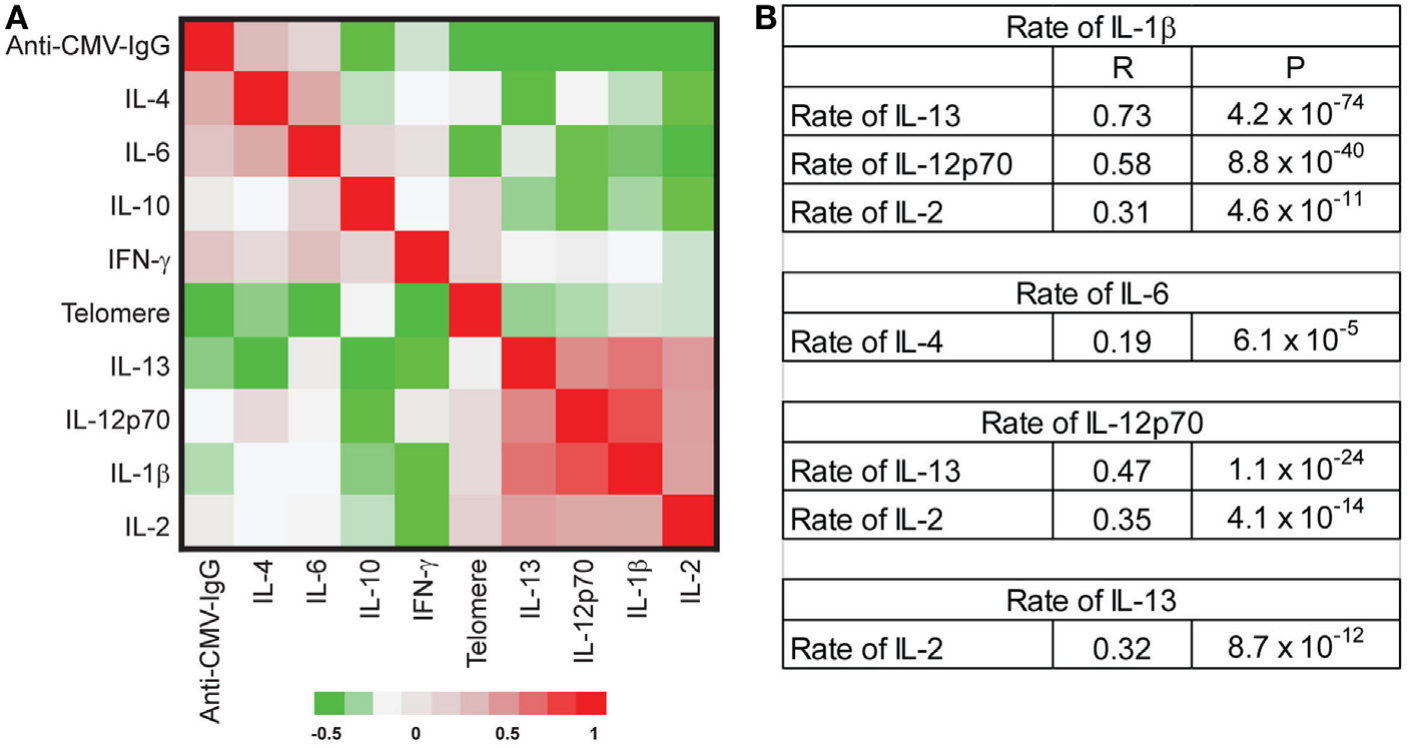
No correlation of the rate changes among telomere length attrition, increased inflammatory-related cytokines, and anti-cytomegalovirus (CMV) IgG. **(A)** Partial correlation coefficient among the rate changes of telomere length, eight inflammatory cytokines [IL-1β, IL-2, IL-4, IL-6, IL-10, IL-12p70, IL-13, and interferon gamma (IFN-γ)], and anti-CMV IgG titers. The correlation coefficients between pair comparison of the rate of change were analyzed with adjustment for age, sex, and date, and data presented as a clustered heat map. The scale is −0.5 to 1. **(B)** Selected significant correlations were presented with correlation coefficient (*R*) and *P* values. After adjusting by FDR, *P* value < 0.001 was significant at *P* < 0.05.

The age-associated changes in telomere attrition, increased circulating inflammatory cytokines (IFN-γ and IL-6), and anti-CMV IgG titer observed in our study cohort showed a high degree of inter-individual variation. To determine whether changes in the age-associated in these parameters occurred in parallel or independently across individuals, we used partial correlations (adjusted for age and sex) to assess correlations in the rates of change. We observed significant positive correlations among the rates of changes in some cytokines including (1) rates of change for IL-1β with IL-13, IL-12p70, and IL-2; (2) rates of change for IL-4 with IL-6 and IL-13; (3) rates of change for IL-12p70 with IL-13 and IL-2, and (4) rates of change for IL-13 and IL-2 (Figure 4). Collectively, these findings suggest that there may be some underlying common mechanisms that drive age-associated changes in pro-inflammatory cytokines.

Surprisingly, we found no significant correlations among telomere attrition, circulating inflammatory cytokines, and anti-CMV IgG titers in their rates of change (Figure 4), suggesting that the age-associated changes of these biomarkers had distinct trajectories and acted independently from each other during aging of an individual. Our findings thus provide evidence that the level and the pace of telomere attrition, increased inflammatory cytokines, and anti-CMV IgG titers are independently determined during an individual’s aging.

## Discussion

Although aging affects multiple organs and tissues, the rates of age-related changes display a remarkable degree of variation within the human population. Our previous longitudinal studies of aging of the immune system assessed immune cell composition (10) and telomere length (17), and demonstrated highly individualized changes. In the current study, we determined that three different age-associated biomarkers (telomere length, circulating IFN-γ and IL-6, and anti-CMV IgG titer) and their changes with age (telomere attrition in PBMCs, increased circulating IFN-γ and IL-6, and increased titers of anti-CMV IgG) are not correlated with one another during aging of an individual and that patterns of change are highly heterogeneous across individuals. Our findings did not show the previously reported (37) correlation between IL-1β levels and telomere attrition, and overall, the results of our longitudinal studies suggest that the manifestations of aging-associated changes in the immune system are multifaceted and exhibit independent trajectories. These findings suggest that there is no dominant integrator among these three classes of age-related change.

PBMCs and serum are the most accessible and commonly used materials in human studies, and it must be recognized that PBMCs contain multiple types of cells in varying proportion and that cytokines in serum derive from multiple potential sources. The observed independent rates of changes in telomere length, inflammatory cytokines, and anti-CMV titers in individuals suggest that these changes reflect differential regulation by intrinsic, e.g., genetic, factors, and/or differential environmental exposures that may impact different cell types resulting in distinct phenotypes. Telomere length attrition is closely related to the history of cell divisions (particularly relevant in lymphocytes), which may be less affected by the level of circulating pro-inflammatory cytokines (produced mainly by myeloid derived cells). Chronic infection of CMV impacts on both lymphoid and innate cells (38, 39). Previous studies report no significant association between the level (not the rate of change) of pro-inflammatory cytokines and serostatus of CMV infection (25, 31). Here, we expand upon these observations by demonstrating that the rates of these three age-associated changes are distinct and independent in individuals as they age. Considering that age-associated changes can take years to emerge, changes in one factor can exert secondary effects on other over time. Thus, our failure to observe correlations between immune system parameters could reflect differential time course of events that are in fact causally related. For example, persistent high level of IFN-γ could enhance proliferation of lymphocytes, which could result in loss of telomeres with a delayed time profile. Regardless, the current findings point to the need for using multiple measurements of these biomarkers to better evaluate biological aging of the immune system.

In contrast to the disassociation among age-related changes in telomere length of PBMCs, circulating inflammatory cytokines, and titer of anti-CMV IgG, the changes of various inflammatory cytokines with age show a number of positive correlations. The rate of increase in IL-6 is positively correlated with the rate of change in IL-4, and the rate of IL-1β is positively correlated with the rates of IL-13, IL-12p70, and IL-2. Although not all these cytokines displayed statistically significant age-associated changes, this suggests that the expression of these multiple pro-inflammatory cytokines may be regulated by common stimulators and/or that these cytokines may regulate one another in an autocrine and paracrine fashion. This mutual enhancement of inflammatory cytokine expression may explain why the increase in pro-inflammatory cytokines with age is rarely limited to a single cytokine. In contrast to the previous report that IL-10 decreases with age, we found that IL-10 was increased with age from the cross-sectional analysis (*P*_C_ = 0.022) but that there was no statistically significance age effect from the longitudinal analysis (*P*_L_ = 0.25) in our study cohort. Further studies will be needed to resolve this difference. A better understanding of the collective impact of the increase of multiple pro-inflammatory cytokines on the immune system in the elderly will come from a systems biology approach with longitudinal follow-up.

An accurate assessment of biological aging requires measuring multiple biomarkers and considering them collectively in order to present a meaningful composite index or profile. Unraveling the interactions underlying the age-associated changes of these biomarkers will greatly enhance our understanding of the aging process and the significance of these biomarkers. The finding of highly individualized changes among multiple biomarkers emphasizes the need to collect comprehensive information reflecting biological age of individuals and providing a basis for practicing precision/personalized medicine.

## Ethics Statement

This study protocol was carried out in accordance with the guidelines of the National Institute on Aging and approved by the Institutional Review Board. Study subjects were participants of the Baltimore Longitudinal Study of Aging (BLSA) and gave written informed consent.

## Author Contributions

AL and HL did experiment and data collection. EM performed statistical analysis of the data. MS and PE assisted the sample and data collection. N-pW, LF, and RH supervised the project and wrote the manuscript with approval from all authors. All authors discussed and reviewed the results of the project.

## Conflict of Interest Statement

The authors declare that the research was conducted in the absence of any commercial or financial relationships that could be construed as a potential conflict of interest.
