# Supplementary material for: Telomere Shortening, Inflammatory Cytokines, and Anti-Cytomegalovirus Antibody Follow Distinct Age-Associated Trajectories in Humans
Source: Front Immunol. 2017 Aug 24;8:1027. doi: 10.3389/fimmu.2017.01027 (PMC5609584; doi:10.3389/fimmu.2017.01027)
Supplement: Supplementary file 1 [file data_sheet_1.docx]

**Telomere shortening, inflammatory cytokines, and anti-CMV antibody follow distinct age-associated trajectories in human individuals**

Ana Lustig^1#^, Hans Liu^1#^, E. Jeffrey Metter^2^, Yang An^3^, Melissa Swaby^4^, Palchamy Elango^4^, Luigi Ferrucci^4^, Richard Hodes^5^, and Nan-ping Weng^1*^

^1^ Laboratory of Molecular Biology and Immunology, National Institute on Aging, National Institutes of Health, Baltimore, Maryland, 21224, USA.

^2^ Department of Neurology, University of Tennessee Health Science Center, Memphis, TN 38163, USA

^3^ Laboratory of Behavioral Neuroscience, National Institute on Aging, National Institutes of Health, Baltimore, Maryland, 21224, USA.

^4^ Translational Gerontology Branch, National Institute on Aging, National Institutes of Health, Baltimore, Maryland, 21224, USA.

^5^ Experimental Immunology Branch, National Cancer Institute, National Institutes of Health, Bethesda, Maryland, 20892, USA.

**Supplemental materials:**

**Fig. S1** Change of selected five cytokines with age *in vivo*.

**Table S1**. Demographics of the study subjects at the first evaluation.

**Table S2**. Mean values of telomere length, cytokines, and anti-CMV IgG in each age group.


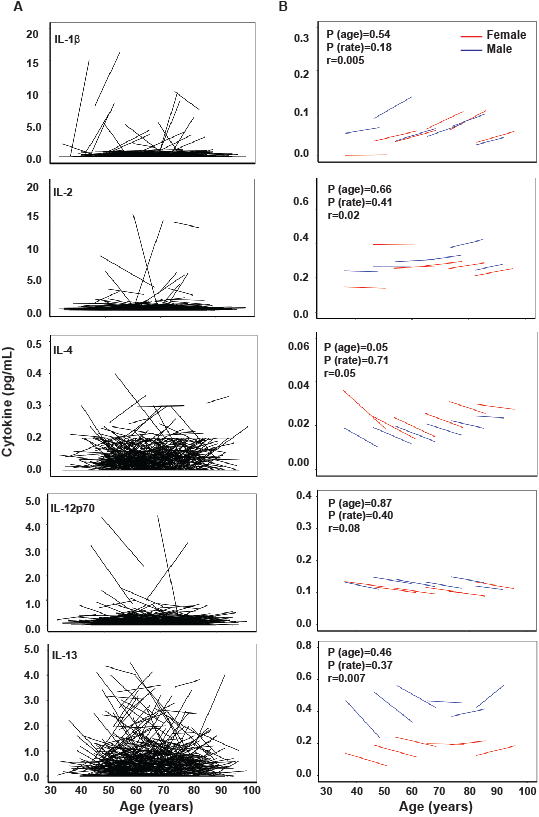


**Fig. S1** Change of selected five cytokines with age *in vivo*. (A) Line graph of IL-1b, IL-2, IL-4, IL12p70 and IL-13 (pg/mL) for the two visits of each subject (N=432). (B Graph of IFN-γ and IL-6 (pg/mL) by five age groups (under 50, 50-59, 60-69,70-79, and 80 years and older) for male in blue and female in red. P values were presented for age and rate as described in Figure 1.

| Table S1: Demographics of Study Participants at First Evaluation | | | |
| --- | --- | --- | --- |
|  | Mean (SD) | Median | Range |
| Sample Size | 456 |  |  |
| Age | 57.4 (13.1) | 56 | 21-88 |
| Female % (N) | 48.5 (226) |  |  |
| Follow-up (years) | 13.3 (3.4) | 14 | 6.9 - 19.2 |
| Education (years) | 17.5 (2.5) | 8 | 8-25 |
| Race (%) |  |  |  |
| White | 78.8 |  |  |
| African American | 15.9 |  |  |
| Other | 5.4 |  |  |
| Weight (kg) | 77.4 (15.8) | 77 | 41.2 – 142.9 |
| Height (cm) | 169.6 (9.4) | 170 | 145.0 – 197.7 |
| BMI | 26.7 (4.3) | 26 | 17.4 – 48.6 |
| Body Fat (kg) | 27.5 (9.1) | 26.4 | 7.7 – 66.1 |
| Telomere length (kb) | 5.65 (1.02) | 5.5 | 3.6 - 11.3 |
| Rate of change telomere length (bp/year) | -15.8 (39.7) | -10.9 | -303.9 – 57.5 |
| WBC | 6053 (1431) | 5900 | 1700 - 11500 |
| CMV (IgG U/mL) | 12.8 (14.7) | 7.9 | 0.0 – 63.5 |
| IFNG (pg/mL) | 6.8 (26.0) | 3.9 | 0 - 505 |
| IL-10 | 0.43 (0.53) | 0.32 | 0.00 – 7.53 |
| IL-12p70 | 0.54 (1.97) | 0.13 | 0.00 – 19.09 |
| IL-13 | 0.79 (1.31) | 0.4 | 0.00 – 13.85 |
| IL-1beta | 0.23 (0.79) | 0.44 | 0.00 – 10.21 |
| IL-2 | 0.62 (3.88) | 0.28 | 0.00 – 77.46 |
| IL-4 | 0.04 (0.04) | 0.03 | 0.00 – 0.30 |
| IL-6 | 0.94 (1.12) | 0.72 | 0.00 – 16.1 |

| Table S2 Mean telomere length, cytokines, and anti-CMV IgG in age groups | | | | | | | | |
| --- | --- | --- | --- | --- | --- | --- | --- | --- |
| Rate of change | Gender | Age group | | | | | | |
|  |  | <40 | | 40-49* | 50-59 | 60-69 | 70-79 | >80 |
| Telomere length (Kb) | Male | 5.9 (1.7) | 5.2 (0.7) | | 5.1 (0.8) | 5.2 (0.9) | 5.3 (0.8) | 4.8 (0.4) |
|  | Female | 6.6 (0.9) | 6.4 (1.0) | | 5.9 (0.9) | 5.9 (0.7) | 5.9 (0.9) | 5.9 (0.9) |
| IFN-g (pg/mL) | Male |  | 7.0 (19.8) | | 4.7 (6.7) | 3.9 (2.3) | 16.1 (73.0) | 5.6 (5.8) |
|  | Female |  | 4.9 (3.3) | | 6.5 (6.6) | 5.5 (3.4) | 11.0 (26.7) | 6.0 (2.9) |
| IL-6 | Male |  | 0.8 (0.6) | | 1.1 (2.0) | 0.9 (1.1) | 1.2 (1.3) | 0.9 (0.4) |
|  | Female |  | 0.7 (0.4) | | 0.8 (0.6) | 1.1 (1.0) | 1.2 (0.8) | 1.3 (0.9) |
| IL-10 | Male |  | 0.35 (0.17) | | 0.42 (0.64) | 0.34 (0.17) | 0.38 (0.42) | 0.36 (0.16) |
|  | Female |  | 0.47 (0.39) | | 0.40 (0.37) | 0.48 (0.24) | 0.85 (1.52) | 0.49 (0.45) |
| Anti-CMV IgG | Male |  | 4.4 (7.8) | | 12.0 (14.7) | 13.2 (15.1) | 15.9 (14.2) | 16.9 (19.0) |
|  | Female |  | 9.9 (14.2) | | 13.0 (15.0) | 14.9 (12.6) | 16.2 (15.8) | 28.1 (18.3) |
| - For the age group of IFN-g, IL-6, IL-10, and anti-CMV IgG include under 40 years old subjects. - Mean (SD) | | | | | | | | |
